# (*E*)-5-Phenyl-*N*-(2-thienylmethyl­ene)-1,3,4-thia­diazole-2-amine

**DOI:** 10.1107/S1600536809023447

**Published:** 2009-06-24

**Authors:** Güneş Demirtaş, Necmi Dege, Memet Şekerci, Süleyman Servi, Muharrem Dinçer

**Affiliations:** a Department of Physics, Faculty of Arts and Sciences, Ondokuz Mayıs University, 55139 Samsun, Turkey; b Department of Chemistry, Faculty of Arts and Sciences, Fırat Universty, 38039 Elazığ, Turkey

## Abstract

In the title compound, C_13_H_9_N_3_S_2_, the thio­phene and phenyl rings are oriented at dihedral angles of 8.00 (7) and 6.31 (7)°, respectively, with respect to the central thia­diazole ring. No significant C—H⋯S and π–π inter­actions exist in the crystal structure.

## Related literature

For the biological activity of [1,3,4]-thia­diazole-containing compounds, see: Foroumadi, Soltani *et al.* (2003 ▶); Foroumadi, Mansouri *et al.* (2003 ▶); Holla *et al.* (2002 ▶); Genc & Servi (2005 ▶); Servi *et al.* (2005 ▶). For a related structure, see: Dege *et al.* (2006 ▶).
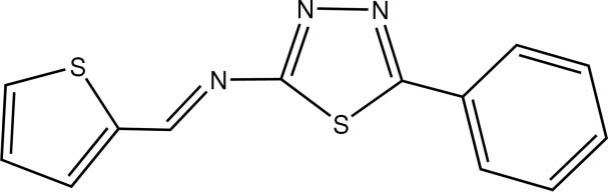


## Experimental

### 

#### Crystal data

C_13_H_9_N_3_S_2_
*M* *_r_* = 271.35
Monoclinic, 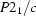

*a* = 6.2238 (3) Å
*b* = 7.7393 (3) Å
*c* = 25.6959 (13) Å
β = 94.701 (4)°
*V* = 1233.55 (10) Å^3^
*Z* = 4
Mo *K*α radiation
μ = 0.41 mm^−1^
*T* = 293 K
0.74 × 0.48 × 0.16 mm

#### Data collection

Stoe IPDS-2 diffractometer
Absorption correction: integration (*X-RED*; Stoe & Cie, 2002 ▶) *T*_min_ = 0.815, *T*_max_ = 0.943
10930 measured reflections
2619 independent reflections
2275 reflections with *I* > 2σ(*I*)
*R*_int_ = 0.020

#### Refinement

*R*[*F* ^2^ > 2σ(*F* ^2^)] = 0.026
*wR*(*F* ^2^) = 0.080
*S* = 1.05
2619 reflections
176 parameters
H atoms treated by a mixture of independent and constrained refinement
Δρ_max_ = 0.18 e Å^−3^
Δρ_min_ = −0.17 e Å^−3^

### 

Data collection: *X-AREA* (Stoe & Cie, 2002 ▶); cell refinement: *X-AREA*; data reduction: *X-RED* (Stoe & Cie, 2002 ▶); program(s) used to solve structure: *SHELXS97* (Sheldrick, 2008 ▶); program(s) used to refine structure: *SHELXL97* (Sheldrick, 2008 ▶); molecular graphics: *ORTEP-3 for Windows* (Farrugia, 1997 ▶); software used to prepare material for publication: *WinGX* (Farrugia, 1999 ▶) and *PLATON* (Spek, 2009 ▶).

## Supplementary Material

Crystal structure: contains datablocks I, global. DOI: 10.1107/S1600536809023447/ci2829sup1.cif

Structure factors: contains datablocks I. DOI: 10.1107/S1600536809023447/ci2829Isup2.hkl

Additional supplementary materials:  crystallographic information; 3D view; checkCIF report

## Figures and Tables

**Fig. 1.**
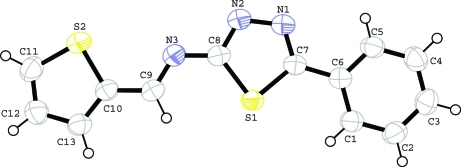
The molecular structure of (I), with the atom-numbering scheme. Displacement ellipsoids are drawn at the 50% probability level.

## supplementary crystallographic information

### Comment

[1,3,4]-Thiadiazoles and their derivatives exhibit diverse biological activites possibly due to the presence of ═N—C—S moiety (Holla *et al.*, 2002; Servi *et al.*, 2005; Genc & Servi, 2005). Various phenyl substituted [1,3,4]-thiazole-2-amines and their derivatives have recently received significant importance because of their diverse biological properties (Foroumadi, Soltani *et al.*, 2003; Foroumadi, Mansouri *et al.*, 2003).We report here the crystal structure of the title compound, (I).

In (I), the C7—S1 [1.7234 (13) Å] distance is shorter than the C8—S1 distance [1.7411 (13) Å]. The C11–S2 distance of 1.6993 (17) Å is shorter the C10—S2 distance of 1.7229 (13) Å and other C—S bonds in the molecule. These bond distances agree well with the corresponding values reported for 1-(thiophen-2-ylmethyl)-2-(thiophen-2-yl)-1*H*- benzimidazole (Dege *et al.*, 2006). The thiophene and phenyl rings are oriented at dihedral angles of 8.00 (7)° and 6.31 (7)°, respectively, with respect to the central thiadiazole ring.

No significant C—H···S and π-π interactions are observed.

### Experimental

A solution of 5-phenyl-1,3,4-thiadiazole-2-amine (0.01 mol) in absolute ethanol (20 ml) was added in small portions to a solution of thiophen-2-carbaldehyde (0.01 mol) in absolute ethanol (30 ml). The reaction mixture was maintained at 343 K for 4 h, cooled and then added to ice-cold water. The resulting solid was washed with water, dried and recrystallized from ethanol (yield 77%). IR (cm^-1^): 3078 (Ar H), 1633 (C═C), 1589 (C═N); ^1^H-NMR: 7.2–7.9 (m, 8H, aromatic protons), 9.2 (s, ^1^H, N═CH proton).

### Refinement

Atoms H1, H9 and H12 were located in a difference map and refined freely. The other H atoms were positioned geometrically and refined using a riding model, with C-H = 0.93 Å and *U*_iso_(H) = 1.2U_eq_(C).

### Crystal data

**Table:**

C_13_H_9_N_3_S_2_	*F*(000) = 560
*M**_r_* = 271.35	*D*_x_ = 1.461 Mg m^−^^3^
Monoclinic, *P*2_1_/*c*	Mo *K*α radiation, λ = 0.71073 Å
Hall symbol: -P 2ybc	Cell parameters from 17067 reflections
*a* = 6.2238 (3) Å	θ = 1.6–26.8°
*b* = 7.7393 (3) Å	µ = 0.41 mm^−^^1^
*c* = 25.6959 (13) Å	*T* = 293 K
β = 94.701 (4)°	Plate, yellow
*V* = 1233.55 (10) Å^3^	0.74 × 0.48 × 0.16 mm
*Z* = 4	

### Data collection

**Table:**

Stoe IPDS-2 diffractometer	2619 independent reflections
Radiation source: fine-focus sealed tube	2275 reflections with *I* > 2σ(*I*)
plane graphite	*R*_int_ = 0.020
Detector resolution: 6.67 pixels mm^-1^	θ_max_ = 26.8°, θ_min_ = 1.6°
ω scans	*h* = −7→7
Absorption correction: integration (*X-RED*; Stoe & Cie, 2002)	*k* = −9→9
*T*_min_ = 0.815, *T*_max_ = 0.943	*l* = −32→32
10930 measured reflections	

### Refinement

**Table:**

Refinement on *F*^2^	Secondary atom site location: difference Fourier map
Least-squares matrix: full	Hydrogen site location: inferred from neighbouring sites
*R*[*F*^2^ > 2σ(*F*^2^)] = 0.026	H atoms treated by a mixture of independent and constrained refinement
*wR*(*F*^2^) = 0.080	*w* = 1/[σ^2^(*F*_o_^2^) + (0.0481*P*)^2^ + 0.1148*P*] where *P* = (*F*_o_^2^ + 2*F*_c_^2^)/3
*S* = 1.05	(Δ/σ)_max_ = 0.001
2619 reflections	Δρ_max_ = 0.18 e Å^−^^3^
176 parameters	Δρ_min_ = −0.17 e Å^−^^3^
0 restraints	Extinction correction: *SHELXL97* (Sheldrick, 2008), Fc^*^=kFc[1+0.001xFc^2^λ^3^/sin(2θ)]^-1/4^
Primary atom site location: structure-invariant direct methods	Extinction coefficient: 0.002634 (11)

### Special details

**Table:**

Geometry. All e.s.d.'s (except the e.s.d. in the dihedral angle between two l.s. planes) are estimated using the full covariance matrix. The cell e.s.d.'s are taken into account individually in the estimation of e.s.d.'s in distances, angles and torsion angles; correlations between e.s.d.'s in cell parameters are only used when they are defined by crystal symmetry. An approximate (isotropic) treatment of cell e.s.d.'s is used for estimating e.s.d.'s involving l.s. planes.
Refinement. Refinement of *F*^2^ against ALL reflections. The weighted *R*-factor *wR* and goodness of fit *S* are based on *F*^2^, conventional *R*-factors *R* are based on *F*, with *F* set to zero for negative *F*^2^. The threshold expression of *F*^2^ > σ(*F*^2^) is used only for calculating *R*-factors(gt) *etc*. and is not relevant to the choice of reflections for refinement. *R*-factors based on *F*^2^ are statistically about twice as large as those based on *F*, and *R*- factors based on ALL data will be even larger.

### Fractional atomic coordinates and isotropic or equivalent isotropic displacement parameters (Å^2^)

**Table:**

	*x*	*y*	*z*	*U*_iso_*/*U*_eq_	
C1	0.0290 (2)	0.1603 (2)	0.57838 (6)	0.0543 (3)	
C2	0.0105 (3)	0.0893 (2)	0.62710 (6)	0.0615 (4)	
H2	0.1311	0.0817	0.6509	0.074*	
C3	−0.1859 (3)	0.0297 (2)	0.64059 (6)	0.0621 (4)	
H3	−0.1976	−0.0190	0.6733	0.075*	
C4	−0.3643 (2)	0.0422 (2)	0.60563 (6)	0.0627 (4)	
H4	−0.4967	0.0014	0.6147	0.075*	
C5	−0.3479 (2)	0.1152 (2)	0.55709 (6)	0.0565 (3)	
H5	−0.4698	0.1247	0.5338	0.068*	
C6	−0.1503 (2)	0.17452 (16)	0.54281 (5)	0.0461 (3)	
C7	−0.13539 (19)	0.24791 (17)	0.49053 (5)	0.0466 (3)	
C8	−0.0473 (2)	0.36506 (18)	0.40911 (5)	0.0500 (3)	
C9	0.2276 (2)	0.46866 (18)	0.36247 (5)	0.0500 (3)	
C10	0.3030 (2)	0.55163 (17)	0.31753 (5)	0.0480 (3)	
C11	0.3279 (3)	0.7018 (2)	0.23422 (6)	0.0643 (4)	
H11	0.3078	0.7541	0.2016	0.077*	
C12	0.5205 (3)	0.6920 (2)	0.26256 (7)	0.0631 (4)	
C13	0.5080 (2)	0.60554 (18)	0.31021 (6)	0.0547 (3)	
H13	0.6259	0.5869	0.3342	0.066*	
N1	−0.30366 (19)	0.27346 (19)	0.45777 (5)	0.0656 (4)	
N2	−0.25288 (19)	0.3405 (2)	0.41099 (5)	0.0674 (4)	
N3	0.02776 (18)	0.43483 (15)	0.36467 (4)	0.0530 (3)	
S1	0.10458 (5)	0.30465 (5)	0.466131 (13)	0.04910 (12)	
S2	0.12673 (6)	0.60757 (5)	0.265024 (14)	0.06061 (14)	
H9	0.333 (3)	0.444 (2)	0.3905 (6)	0.065 (4)*	
H1	0.158 (3)	0.206 (2)	0.5694 (7)	0.071 (5)*	
H12	0.645 (3)	0.736 (3)	0.2497 (8)	0.086 (6)*	

### Atomic displacement parameters (Å^2^)

**Table:**

	*U*^11^	*U*^22^	*U*^33^	*U*^12^	*U*^13^	*U*^23^
C1	0.0472 (7)	0.0616 (8)	0.0537 (7)	−0.0008 (6)	0.0013 (6)	−0.0007 (6)
C2	0.0579 (8)	0.0733 (10)	0.0520 (8)	0.0033 (7)	−0.0038 (6)	0.0016 (7)
C3	0.0714 (9)	0.0656 (9)	0.0502 (8)	0.0040 (7)	0.0103 (7)	0.0006 (7)
C4	0.0557 (8)	0.0725 (10)	0.0617 (9)	−0.0027 (7)	0.0151 (7)	−0.0015 (7)
C5	0.0455 (7)	0.0690 (9)	0.0549 (8)	0.0005 (6)	0.0033 (6)	−0.0047 (7)
C6	0.0460 (6)	0.0435 (7)	0.0486 (6)	0.0036 (5)	0.0032 (5)	−0.0061 (5)
C7	0.0425 (6)	0.0452 (6)	0.0516 (7)	0.0016 (5)	0.0007 (5)	−0.0035 (5)
C8	0.0466 (7)	0.0514 (7)	0.0509 (7)	0.0011 (5)	−0.0026 (5)	0.0001 (6)
C9	0.0513 (7)	0.0494 (7)	0.0483 (7)	0.0046 (6)	−0.0021 (5)	−0.0003 (6)
C10	0.0496 (7)	0.0469 (7)	0.0467 (7)	0.0051 (5)	−0.0010 (5)	−0.0019 (5)
C11	0.0729 (10)	0.0655 (9)	0.0537 (8)	−0.0013 (7)	0.0004 (7)	0.0108 (7)
C12	0.0590 (8)	0.0636 (10)	0.0670 (9)	−0.0037 (7)	0.0073 (7)	0.0085 (7)
C13	0.0503 (7)	0.0552 (8)	0.0576 (8)	0.0031 (6)	−0.0020 (6)	0.0031 (6)
N1	0.0443 (6)	0.0864 (9)	0.0647 (8)	−0.0049 (6)	−0.0039 (5)	0.0167 (7)
N2	0.0479 (6)	0.0901 (10)	0.0622 (7)	−0.0051 (6)	−0.0068 (5)	0.0208 (7)
N3	0.0518 (6)	0.0564 (7)	0.0499 (6)	−0.0003 (5)	−0.0009 (5)	0.0045 (5)
S1	0.04071 (18)	0.0581 (2)	0.04783 (19)	0.00331 (13)	−0.00016 (12)	0.00054 (14)
S2	0.0538 (2)	0.0724 (3)	0.0536 (2)	−0.00062 (16)	−0.00781 (15)	0.00514 (17)

### Geometric parameters (Å, °)

**Table:**

C1—C2	1.381 (2)	C8—N3	1.3787 (18)
C1—C6	1.3875 (19)	C8—S1	1.7411 (13)
C1—H1	0.923 (18)	C9—N3	1.2770 (18)
C2—C3	1.377 (2)	C9—C10	1.4335 (19)
C2—H2	0.93	C9—H9	0.952 (16)
C3—C4	1.374 (2)	C10—C13	1.3697 (19)
C3—H3	0.93	C10—S2	1.7229 (13)
C4—C5	1.380 (2)	C11—C12	1.353 (2)
C4—H4	0.93	C11—S2	1.6993 (17)
C5—C6	1.3900 (19)	C11—H11	0.93
C5—H5	0.93	C12—C13	1.403 (2)
C6—C7	1.4684 (19)	C12—H12	0.929 (19)
C7—N1	1.3039 (17)	C13—H13	0.93
C7—S1	1.7234 (13)	N1—N2	1.3696 (19)
C8—N2	1.2987 (18)		
			
C2—C1—C6	120.44 (14)	N2—C8—S1	113.44 (11)
C2—C1—H1	121.1 (12)	N3—C8—S1	127.22 (10)
C6—C1—H1	118.3 (11)	N3—C9—C10	120.78 (12)
C3—C2—C1	120.20 (14)	N3—C9—H9	122.7 (10)
C3—C2—H2	119.9	C10—C9—H9	116.5 (10)
C1—C2—H2	119.9	C13—C10—C9	128.09 (12)
C4—C3—C2	119.90 (14)	C13—C10—S2	110.88 (10)
C4—C3—H3	120.0	C9—C10—S2	120.94 (10)
C2—C3—H3	120.0	C12—C11—S2	112.30 (12)
C3—C4—C5	120.28 (14)	C12—C11—H11	123.9
C3—C4—H4	119.9	S2—C11—H11	123.9
C5—C4—H4	119.9	C11—C12—C13	112.73 (14)
C4—C5—C6	120.40 (14)	C11—C12—H12	121.0 (13)
C4—C5—H5	119.8	C13—C12—H12	126.3 (13)
C6—C5—H5	119.8	C10—C13—C12	112.57 (13)
C1—C6—C5	118.77 (13)	C10—C13—H13	123.7
C1—C6—C7	121.70 (12)	C12—C13—H13	123.7
C5—C6—C7	119.53 (12)	C7—N1—N2	113.15 (12)
N1—C7—C6	122.79 (12)	C8—N2—N1	112.78 (12)
N1—C7—S1	113.62 (11)	C9—N3—C8	120.94 (12)
C6—C7—S1	123.58 (9)	C7—S1—C8	87.01 (6)
N2—C8—N3	119.33 (12)	C11—S2—C10	91.51 (7)
			
C6—C1—C2—C3	0.9 (2)	C11—C12—C13—C10	−0.5 (2)
C1—C2—C3—C4	−0.5 (2)	C6—C7—N1—N2	−179.04 (13)
C2—C3—C4—C5	−0.3 (2)	S1—C7—N1—N2	−0.15 (18)
C3—C4—C5—C6	0.9 (2)	N3—C8—N2—N1	−178.89 (13)
C2—C1—C6—C5	−0.3 (2)	S1—C8—N2—N1	0.45 (19)
C2—C1—C6—C7	−179.40 (13)	C7—N1—N2—C8	−0.2 (2)
C4—C5—C6—C1	−0.6 (2)	C10—C9—N3—C8	−175.86 (12)
C4—C5—C6—C7	178.55 (14)	N2—C8—N3—C9	176.50 (15)
C1—C6—C7—N1	−175.17 (14)	S1—C8—N3—C9	−2.7 (2)
C5—C6—C7—N1	5.7 (2)	N1—C7—S1—C8	0.32 (12)
C1—C6—C7—S1	6.05 (19)	C6—C7—S1—C8	179.20 (12)
C5—C6—C7—S1	−173.02 (11)	N2—C8—S1—C7	−0.44 (13)
N3—C9—C10—C13	175.89 (14)	N3—C8—S1—C7	178.84 (13)
N3—C9—C10—S2	−0.24 (19)	C12—C11—S2—C10	−0.24 (14)
S2—C11—C12—C13	0.5 (2)	C13—C10—S2—C11	−0.04 (12)
C9—C10—C13—C12	−176.14 (14)	C9—C10—S2—C11	176.70 (12)
S2—C10—C13—C12	0.30 (17)		
